# Host Functional Response to a Prototypic Orally Delivered Self-Replicating Vaccine Platform

**DOI:** 10.3390/vaccines12070701

**Published:** 2024-06-21

**Authors:** Allison C. Vilander, Julia Burak, Darby Gilfillan, Gregg A. Dean, Zaid Abdo

**Affiliations:** 1 Department of Microbiology, Immunology and Pathology, College of Veterinary Medicine and Biomedical Sciences, Colorado State University, Fort Collins, CO 80521, USA; allison.vilander@colostate.edu (A.C.V.); darby.gilfillan@colostate.edu (D.G.); 2 Department of Clinical Science, College of Veterinary Medicine and Biomedical Sciences, Colorado State University, Fort Collins, CO 80521, USA; julia.burak@colostate.edu

**Keywords:** systems vaccinology, *Lactobacillus acidophilus*, mucosal vaccines, transcriptomics

## Abstract

The development of mucosal vaccines has been limited and could be aided by a systems vaccinology approach to identify platforms and adjuvant strategies that induce protective immune responses. The induction of local immune responses by mucosal-delivered vaccines has been difficult to evaluate from peripheral samples, as systemic responses often do not correlate with the mucosal response. Here, we utilized transcriptomics in combination with Gene Set Enrichment Analysis (GSEA) to assess innate immune activation by an oral probiotic *Lactobacillus acidophilus*-based vaccine platform in mice. The goal was to explore the earliest immune responses elicited after oral immunization at the Peyer’s patch. Twenty-four hours after oral delivery of the *L. acidophilus* vaccine platform, we found an abundance of *L. acidophilus* at Peyer’s patches and detected expression of the vaccine viral proteins and adjuvants, confirming in vivo vaccine delivery. Compared to mice orally dosed with buffer or wild-type *L. acidophilus*, we identified enhanced responses in immune pathways related to cytokine and gene signaling, T and B cell activation, phagocytosis, and humoral responses. While more work is needed to correlate these pathways with protection from infection and/or disease, they indicate this method’s potential to evaluate and aid in the iterative development of next-generation mucosal vaccines.

## 1. Introduction

Enteric pathogens remain an important global cause of morbidity and mortality, particularly in children <5 years of age. The mucosal immune system is vast and highly capable of preventing or controlling local infections, and it is clear that immunization by a mucosal route can effectively induce systemic immune responses [1]. Parenteral immunization, in contrast, rarely induces a robust mucosal immune response [2]. Licensed orally delivered vaccines exist against vibrio, polio, rotavirus, salmonella, and adenovirus (not all are available to the general public), and all are either whole-inactivated or live-attenuated [2]. However, these vaccines are often less effective in low- and middle-income countries (LMICs) [3]. As such, it is broadly acknowledged that new mucosal subunit vaccine platforms are needed for enteric disease prevention in LMICs. The development of new mucosal vaccines would benefit from a systems vaccinology approach to predict and identify strategies that could prove immunogenic in the mucosal environment. The use of these approaches to aid in the design of mucosal vaccines has been underutilized, especially because mucosal surfaces and immune inductive sites are difficult to access without invasive sampling procedures.

Probiotic organisms as adjuvants and vaccine platforms for mucosal immunization are of growing interest because probiotic bacteria engage the host immune system naturally via microbe-associated molecular patterns (MAMPs), and some probiotic organisms have evolved to occupy a niche within the intestine that allows access to mucosal immune inductive sites [4,5]. A probiotic platform offers a self-replicating vector that can be orally delivered, inexpensively produced, and customized to induce and shape the adaptive immune response through targeted innate and adaptive immune activation. Probiotic use as a vaccine platform requires an understanding of the balance between immune tolerance and activation, which is an area where knowledge gaps remain. Unlike systemic immunization, where the baseline status is immune readiness, the bias at mucosal surfaces is toward tolerance (reviewed in [6,7]). Given that mucosal surfaces are constantly exposed to a variety of MAMPs that can activate innate immune responses through pattern recognition receptors (PRRs), it is not surprising that tipping the balance from tolerance to immune activation requires multiple activation signals and pathways in the context of each other and at specific locations within the mucosal tissues to drive immune recognition. This is why, without question, adjuvants are key to our ability to predictably induce protective immune responses against enteric pathogens.

We have explored the immune-activating characteristics of the probiotic bacterium *Lactobacillus acidophilus*, which are mediated through DC-SIGN, TLR2, and NOD2 [8,9,10]. We have further demonstrated enhanced antigen-specific immune induction using recombinant *Lactobacillus acidophilus* expressing a variety of antigens (e.g., HIV, rotavirus, and ovalbumin) and either FliC, a component of bacterial flagella on *Salmonella enterica* serovar Typhimurium (a TLR5 agonist), or *E. coli* type I pilus microfold cell targeting adhesion protein, FimH (for M cell targeting and TLR4 activation) [11,12,13,14]. We have used these constructs to interrogate the individual mechanisms of action required to induce mucosal and systemic immune responses. However, this targeted and empirical approach to vaccine development is notoriously slow, and the mechanisms of action of combinations of MAMPs, along with M-cell targeting, have not been studied. Here, we report an innovative application of systems vaccinology to accelerate the development of *L. acidophilus* as an oral vaccine platform. We utilized transcriptomics to assess the in vivo performance of the rLA vaccine platform in combination with Gene Set Enrichment Analysis (GSEA) to explore the earliest immune responses elicited after oral immunization at hard-to-access mucosal immune inductive sites.

## 2. Materials and Methods

### 2.1. Animal Ethics Statement

This study was carried out in strict accordance with relevant guidelines and regulations, including the ARRIVE guidelines (https://arriveguidelines.org, accessed 1 October 2022), the Guide for the Care and Use of Laboratory Animals of the National Institutes of Health, and the Association for the Assessment of Laboratory Animal Care standard, with approval from the Institutional Animal Care and Use Committee of Colorado State University (protocol number 18-1234A, 29 November 2022). Animals were monitored daily for clinical signs of illness or stress and humanely euthanized at the study endpoint via carbon dioxide inhalation and exsanguination by heart puncture.

### 2.2. Experimental Design

We used three vaccine treatments to evaluate the host’s response to oral vaccination at a mucosal inductive site. We used our recombinant NCK56 *Lactobacillus acidophilus* probiotic vaccine platform expressing two rotavirus antigens, VP8-1 protein and VP8 10 amino acid peptide (VP8Pep), in combination with dual-adjuvants, *E. coli* type I pilus microfold cell targeting adhesion protein, FimH, and FliC, a component of bacterial flagella on *Salmonella enterica* serovar Typhimurium (GAD85), as our first treatment, as described in detail in [14]. Controls included the NCK56 *Lactobacillus acidophilus* wild-type strain expressing neither antigens nor adjuvants (NCK56, positive control) and a no-bacteria negative control with only the soybean trypsin inhibitor (Sigma-Aldrich) buffer (STI) used to resuspend all the probiotic strains. Eighteen 6- to 8-week-old C57BL/6J mice (Jackson Laboratories, Bar Harbor, ME, USA) were assigned equally (six mice, three male and three female) to each treatment group. One female mouse died during the experiment, resulting in the STI group having five mice (3 male and 2 female) for a total of 17 mice post-completion (Table 1). Additionally, one of the GAD85 samples, Male V1, had low sequence depth and did not reflect the current structure of the community, and hence was dropped from all subsequent analyses. Mice were maintained in specific pathogen-free conditions, housed socially in single sex groups (2–3 mice per cage) in commercially available individually ventilated cages, and provided with autoclaved bedding and enrichment in accordance with the animal care guidelines at Colorado State University. Animals were fed ad libitum commercially irradiated rodent chow (Teklad Global, Envigo, Indianapolis, IN, USA) and tap water filtered via reverse osmosis in autoclaved water bottles.

### 2.3. Treatment Preparation and Immunization

The GAD85 and NCK56 strains were grown at 37 °C overnight in MRS broth (BD Diagnostics, Sparks, MD, USA). The FliC protein is expressed from a plasmid that confers erythromycin resistance, and the MRS medium was supplemented with 5 µg/mL erythromycin (Ery; Teknova, Hollister, CA, USA). Overnight cultures were diluted 1:10 and grown to an exponential level for 90 min, then washed twice in 1xPBS (Corning, Corning, NY, USA). GAD85 and NCK56 strains were individually resuspended to a concentration of 5 × 10^9^ CFU per 200 µL of soybean trypsin inhibitor (Sigma-Aldrich Inc., St. Louis, MO, USA) buffer containing 100 µm sodium bicarbonate (NaHCO_3_). Colony-forming units (CFUs) were calculated using an optical density of 600nm. All mice were immunized by oral gavage with 200 µL of either GAD85, NCK56, or soybean trypsin inhibitor buffer (STI) alone at 24 h and 1 h prior to necropsy and sample collection. The rationale for the immunization strategy was to (1) allow time for the expression of inducible host genes within Peyer’s patches and (2) ensure GAD85 and NCK56 were present in the lumen of the small intestine to allow metatranscriptomic analysis under in vivo conditions. We have previously shown that recombinant lactobacilli are mostly cleared from the host within 24 h and do not colonize the small intestine (10.1128/CVI.05277-11; Stoeker et al.) [15].

### 2.4. Sample Collection

The entire small intestine was initially collected in RPMI (Corning) media supplemented with 0.1% gentamicin (Sigma-Aldrich) and 1% penicillin/streptomycin (Caisson Labs, Smithfield, UT, USA). A section of the ileum containing a single Peyer’s patch closest to the cecum, along with any digestive content present, was cut using a sterile razor blade and added to ten times the tissue weight in microliters of DNA/RNA Shield (Zymo Research, Irvine, CA, USA). Peyer’s patches were identified grossly and sampled so that the entire Peyer’s patch was collected. The weights of the collected tissue ranged from 18 to 30 mg. Tissue slices were stored at −80 °C until nucleic acid extraction, and the entire collected section was used.

### 2.5. Nucleic Acid Extraction and Sequencing 

DNA and RNA were extracted from the tissue slices containing Peyer’s patches and digestive content using the Zymo Quick-DNA/RNA Miniprep Plus 50-prep Kit (Zymo Research Corporation, Irvine, CA, USA), following the manufacturer’s instructions. Briefly, samples were homogenized by bead beating for 60 s in 2 mL tubes (Zymo Research Corporation, Irvine, CA, USA) using an MP Bio FastPrep-24 high-speed homogenizer. The suggested DNase I step was completed. Samples were eluted in 100 µL of the nuclease-free water included with the kit. DNA and RNA quantification were completed using a Qubit 4 fluorometer following the manufacturer’s instructions for the 1× dsDNA High Sensitivity and RNA Broad Range kits (both ThermoFisher Scientific, Waltham, MA, USA), respectively. The extracted DNA and RNA samples were stored at −80 °C and shipped frozen for sequencing. Novogene (Novogene Corporation Inc., Sacramento, CA, USA) performed metagenomic sequencing with a target of 6 Gb (20 M, 2 × 150 paired-end reads) per sample and transcriptomic/metatranscriptomic sequencing with a target of 12 Gb (40 M, 2 × 150 paired-end reads) per sample. All sequencing used an Illumina NovaSeq 6000 and employed Novogene’s and Illumina’s standard library preparation procedures.

### 2.6. Data Processing 

Raw shotgun metagenomics and shotgun transcriptomics/metatranscriptomics sequencing reads, provided by Novogene, were assessed for quality using FastQC v.0.11.9 [16] and MultiQC v1.13.dev0 [17]. Sequencing adapters (5′: AGATCGGAAGAGCGTCGTGTAGGGAAAGAGTGTAGATCTCGGTGGTCGCCGTATCATT and 3′: GATCGGAAGAGCACACGTCTGAACTCCAGTCACGGATGACTATCTCGTATGCCGTCTTCTGCTTG) were removed, and the resulting sequences were quality trimmed using trimmomatic v.0.39 [18] with the ILLUMINACLIP:adapters:2:30:10:2:true SLIDINGWINDOW:4:25 MINLEN:70 parameters.

#### 2.6.1. Metagenomics Processing

Reads were aligned to the C57BL/6J reference genome (GRCm39, RefSeq assembly accession GCF_000001635.27) using the Burrows–Wheeler Aligner (BWA, v.0.7.17-r1188) to remove the murine host genomic reads [19]. Unmapped reads were separated using samtools v1.18 [20] and then classified, utilizing Kraken2 [21], using a custom database that included the mouse reference genome and the bacterial database with goal of assessing the presence and differential abundance of *Lactobacillus acidophilus*.

#### 2.6.2. Transcriptomics/Metatranscriptomics Processing

We used STAR [22], utilizing default parameters, to align the metatranscriptomics sequence reads to the C57BL/6J reference genome (GRCm39, RefSeq assembly accession GCF_000001635.27). This served to both assess the host’s expression in response to the treatments and also separate host sequence reads from the microbiome. The resulting count matrix was generated using HTSeq [23] with the strand-specific parameter -s no and using default parameters otherwise. This count matrix was then exported to R for further analysis utilizing DESeq2 [24]. Unmapped sequence data after STAR alignment were mapped to the mouse genome and, after that, the human genome to remove any sequence reads that might have still associated with these irrelevant genomes. The resulting metatranscriptomics reads were then mapped to the reference DNA sequences associated with the antigens VP8-1 and VP8Pep and the adjuvants FliC and FimH using Geneious Prime v.2023.3.1 (https://www.geneious.com, accessed 1 November 2023).

### 2.7. Statistical Analyses

#### 2.7.1. Metaomics

Metagenomics analysis was only used to confirm the presence of recombinant *Lactobacillus acidophilus* (GAD85). The presence and dominance of *L. acidophilus* in both the GAD85 and NCK56 treatments was assessed utilizing bar plots of the relative abundance tables from Kraken2 [21] using ggplot2 [25] and a custom R script. Metatranscriptomics analysis was also only used to confirm the expression of the antigens and adjuvants associated with GAD85. ggplot2 [25] in R [26] was used to create pile-up plots, restricting the matched mapping to the region of the DNA sequences representing the antigens and adjuvants under study.

#### 2.7.2. Transcriptomics

The expression count tables generated per sample using HTSeq [23] were combined, and per-gene expression data of the mouse transcriptome were compared utilizing DESeq2 using their internal normalization method. Comparisons were made between treatment levels, between treatment levels within mouse sex, and between mouse sex within each treatment level. The resulting tables from these analyses were utilized, along with the normalized data, in the pathway enrichment analyses described below.

Pathway enrichment analysis was performed using GSEA 4.3.2 software. GSEA Standard analysis was performed utilizing the signal2noise ranking metric. The m5:go:bp.2022.1.mm.symbols.gmt gene set from MSigDB was used as the gene set database, and the “Collapse” function was employed using the Mouse_Gene_Symbol_Remapping_MSigDB.v2022.1.Mm.chip file to standardize gene identifiers. The minimum gene set size for the analysis was set to 15, and the maximum gene set size was set to 200. Data visualization was conducted using the Enrichment Map application in Cytoscape v3.9.1 software with Jaccard overlap combined coefficient > 0.375 with combined constant = 0.5. Our goal in conducting these analyses was exploratory due to the small sample size; thus, we set the significance threshold to a discovery level of FDR Q-value ≤ 0.25, with our focus on detecting potential patterns of gene set expression.

Clusters of nodes were grouped using the AutoAnnotate application, utilizing the MCL cluster algorithm. These cluster annotations were manually updated for clarity. Each node shows a unique MSigDB gene set from the M5 GO:BP sub-collection, derived from the biological process (BP) root ontology of the Gene Ontology (GO) collection. Node labels from MSigDB gene set names were manually adjusted for ease of interpretation (proper case, underscore to space, etc.). A legend was manually added at the bottom of the figure using the template provided by the Bader Lab at http://baderlab.org/Software/EnrichmentMap#Legends (accessed 1 November 2023).

## 3. Results

### 3.1. L. acidophilus Dominated the Microbial Community Structure of GAD85- and NCK56-Treated Mice

**Figure 1:**
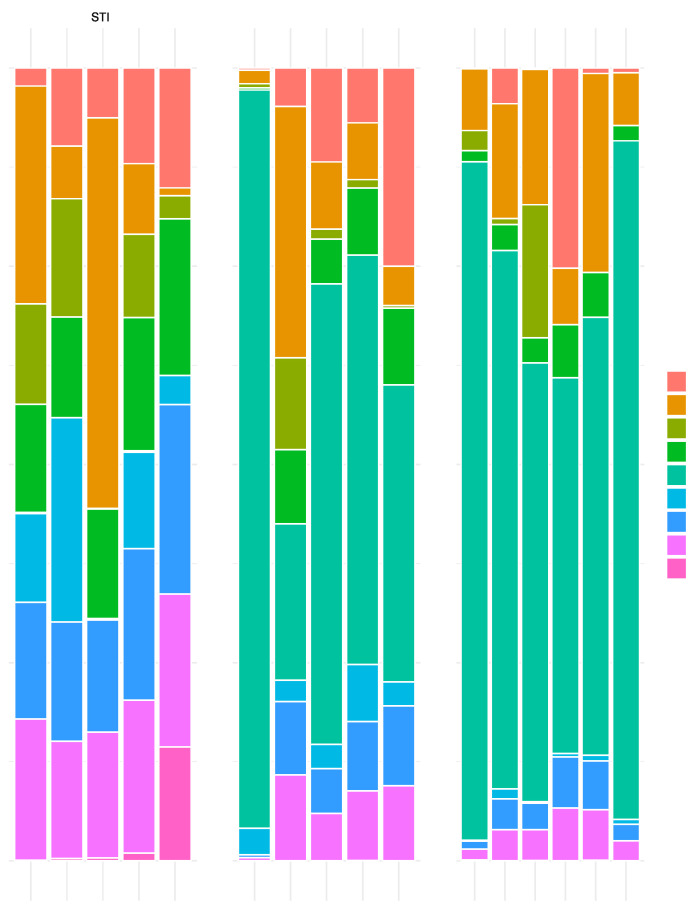
Bar plots showing the proportion of bacterial species (Y-axes) in samples from individual animals (X-axes) for each treatment (STI, GAD85, and NCK56). Only species attaining a proportion of 0.05 or more of the normalized abundance per individual sample are shown in the graph. Each colored segment of the bar per individual identifies the proportion of the species with that color described in the key on the right hand side of the plot. A clear dominance of *L. acidophilus* is observed in the GAD85 and NCK56 treatments.

Metagenomic analysis of ileum samples that included luminal contents revealed the dominance of *L. acidophilus* in each individual animal treated with NCK56 or GAD85 (Figure 1). This dominance of abundance of *L. acidophilus* was expected, given that sampling occurred one hour after the second oral immunization, and demonstrates that the inoculum dose was sufficient to provide relative high levels of exposure at immune inductive sites (Peyer’s patches) in the small intestine.

### 3.2. GAD85 Expresses the VP8 Antigens and the FimH and FliC Adjuvants

**Figure 2:**
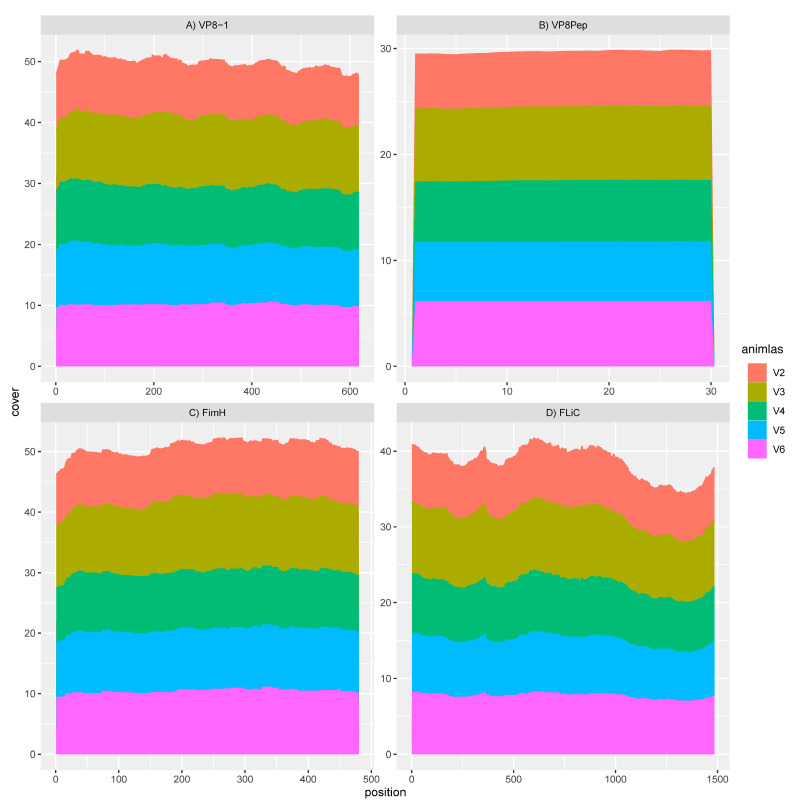
RNA sequence mapping of vaccine antigens and adjuvants in animals treated with GAD85. Gene expression observed in individual animals (V2–V6) for (**A**) the VP8-1 antigen; (**B**) the VP8pep peptide; (**C**) the FimH adjuvant; and (**D**) the FliC adjuvant within the sampled intestine at Peyer’s patches, confirming the function of the rLA vaccine platform. The X-axes of these panels describe the base pair position of the reference sequence of each of these expressed genes, starting at position 0 and ending at the length of that gene, and the Y-axes describe the cumulative read cover that aligns to these genes.

Mapping of the rotavirus antigens and FliC and FimH adjuvants within sample transcriptomes of the GAD85-treated mice, normalized by depth of microbiome sequencing per sample, clearly shows that the VP8-1 protein (Figure 2A), VP8pep (Figure 2B), adjuvant FimH (Figure 2C), and adjuvant FliC (Figure 2D) are being expressed by the vaccine construct. Taken together with the previous section, these results confirm the presence and function of our recombinant *L. acidophilus* (GAD85) vaccine in vivo at sites that induce the immune response.

### 3.3. Immunologically Relevant Gene Sets Were Enriched in GAD85 Compared to NCK56

A total of 1594/3405 gene sets were found to be upregulated in the GAD85 group (Table S1) compared to 1811/3405 in the NCK56 control group (Table S2). At an FDR Q-value ≤ 0.25, there were 163 gene sets enriched in the GAD85 group and 175 in the NCK56 group. At an FDR Q-value ≤ 0.05, there were 16 gene sets enriched in the GAD85 group and 1 in the NCK56 group.

**Figure 3:**
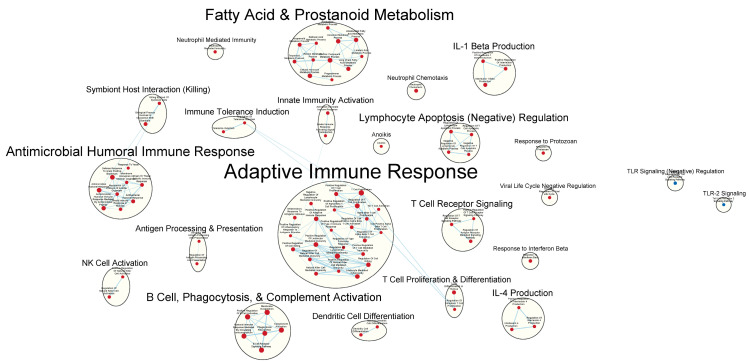
Immunologically relevant gene sets enriched in GAD85 compared to NCK56 at an FDR ≤ 0.25. Nodes represent pathways, and they are grouped within clusters based on closeness of function and shared genes represented by edges linking these nodes. This figure provides an overview of the many immunologically relevant gene sets involved in both innate and adaptive immune responses that were enriched in the GAD85 group compared to the NCK56, positive control, group, as reflected by the cluster labels. Details of the within-cluster pathways, genes, FDR Q-values, and other information can be found in Table S3 and Data S1.

**Figure 4:**
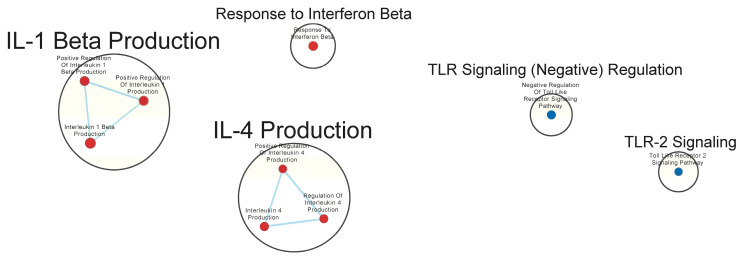
Gene sets related to cytokines and immune signaling enriched in GAD85 compared to NCK56 at an FDR ≤ 0.25. Nodes represent pathways, and they are grouped within clusters based on closeness of function and shared genes represented by edges linking these nodes. This figure highlights the enriched gene sets involved in immune signaling, including those related to IL-1β, IL-4, and IFN-β described in the cluster labels. Details of the within-cluster pathways, genes, FDR Q-values, and other information can be found in Table S3 and Data S1.

**Figure 5:**
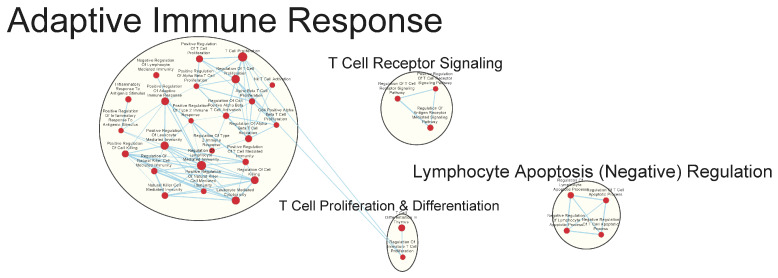
Gene sets related to T cells enriched in GAD85 compared to NCK56 at an FDR ≤ 0.25. Nodes represent pathways, and they are grouped within clusters based on closeness of function and shared genes represented by edges linking these nodes. This figure highlights T cell proliferation and activation pathways. Details of the within-cluster pathways, genes, FDR Q-values, and other information can be found in Table S3 and Data S1.

**Figure 6:**
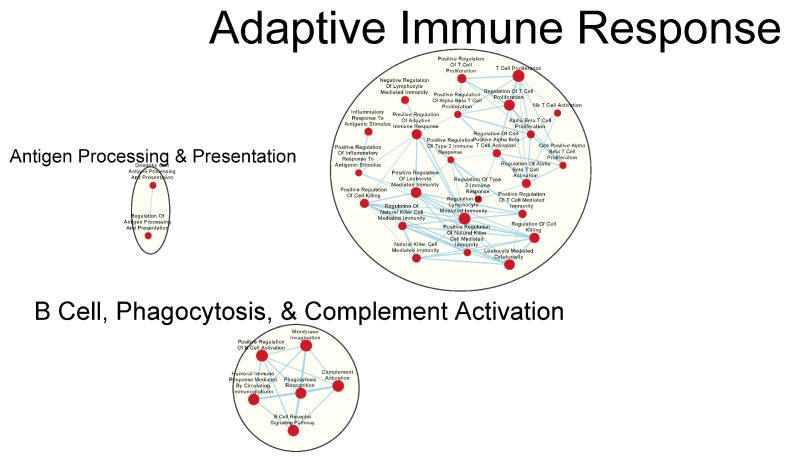
Gene sets related to B cells enriched in GAD85 compared to NCK56 at an FDR ≤ 0.25. Nodes represent pathways, and they are grouped within clusters based on closeness of function and shared genes represented by edges linking these nodes. Details of the within-cluster pathways, genes, FDR Q-values, and other information can be found in Table S3 and Data S1.

Many immunologically relevant gene sets involved in both innate and adaptive immune responses were enriched in the GAD85 group at the FDR Q-value ≤ 0.25 as compared to NCK56 (Figure 3 and Table S3). Enriched gene sets involved in immune signaling included those related to IL-1β, IL-4, and IFN-β (Figure 4 and Table S3). Other innate immune gene sets noted to be upregulated include neutrophil chemotaxis, neutrophil-mediated immunity, and natural killer (NK) cell activation. Bridging the innate and adaptive responses, gene sets for antigen-presenting cell differentiation and antigen processing and presentation were enriched. Cell-mediated immunity gene sets were also identified (Figure S1 and Table S3); T cell responses included proliferation, differentiation, activation, and signaling, as well as upregulation of T cell-mediated immunity, downregulation of T cell apoptosis, and the type 2 immune response. The enrichment of gene sets related to the proliferation and activation of the CD4+ T cell subset was also noted (Figure 5 and Table S3). B cell activation, B cell receptor (BCR) signaling, and the humoral immunoglobulin response gene sets were identified (Figure 6 and Table S3). Also of note were enriched gene sets related to anoikis, antimicrobial activity, viral life cycle regulation, immune tolerance, and the organ/tissue-specific immune response (Figure 3 and Table S3).

The NCK56 group had far fewer enriched gene sets than GAD85, with relevant upregulated gene sets at the FDR Q-value ≤ 0.25, including those related to TLR2 signaling and general regulation of TLR signaling (Figure 4).

### 3.4. Similar Gene Sets Were Enriched in GAD85 and NCK56 When Compared to the STI Control

Many of the same immunologically relevant gene sets were enriched in both the GAD85 and NCK56 treatment groups at the FDR Q-value ≤ 0.25 when compared to the STI group (Table S4 and Figure S2 pertaining to GAD85 vs. STI; Table S5 and Figure S3 for NCK56 vs. STI). There were a total of 2752/3405 gene sets upregulated in the GAD85 group (Table S6) compared to 653/3405 in the STI negative control group (Table S7). At an FDR Q-value ≤ 0.25, gene sets found to be enriched totaled 2329 in the GAD85 group and 43 in the STI group, while at an FDR Q-value ≤ 0.05, there were 1798 gene sets enriched in GAD85 and 11 in STI. A total of 2858/3405 gene sets were upregulated in the NCK56 group (Table S8) compared to 547/3405 in the STI group (Table S9), indicating that the presence of *L. acidophilus* alone initiates a robust immune response. There were 2514 gene sets found to be enriched in the NCK56 group and 45 in the STI group at an FDR Q-value ≤ 0.25, while 1251 gene sets were found to be enriched in the NCK56 group and 13 in the STI group at an FDR Q-value ≤ 0.05.

**Figure 7:**
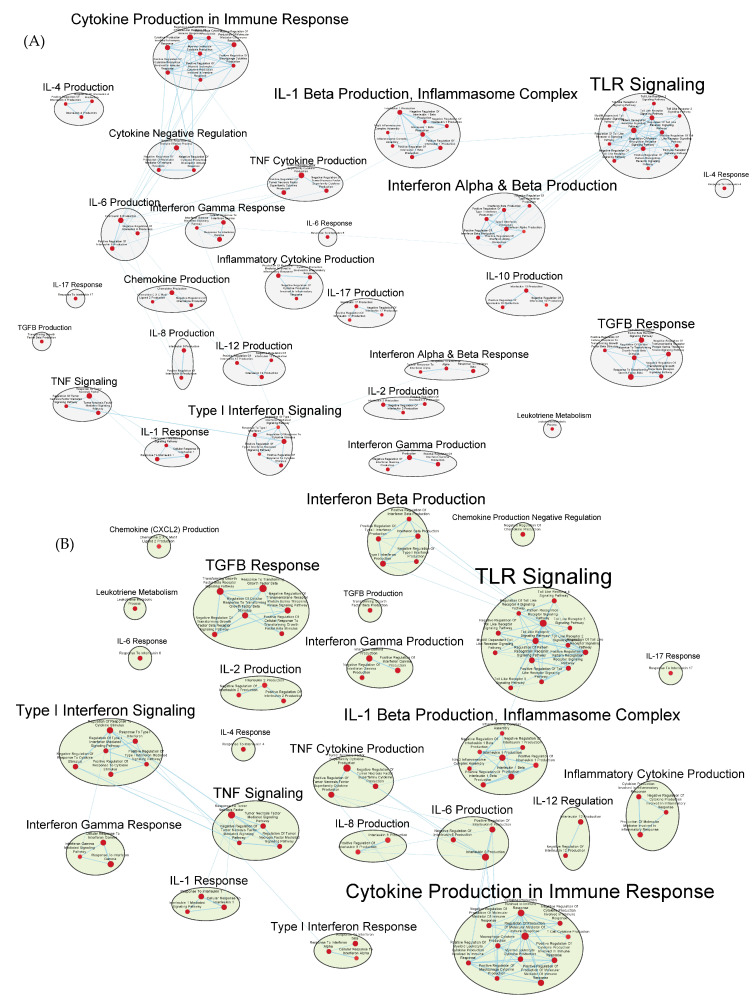
Gene sets related to cytokines and immune signaling enriched in GAD85 compared to STI (**A**) and in NCK56 compared to STI (**B**) at an FDR ≤ 0.25. Nodes represent pathways, and they are grouped within clusters based on closeness of function and shared genes represented by edges linking these nodes. Details of the within-cluster pathways, genes, FDR Q-values, and other information can be found in Tables S4 and S5 for A and B, respectively, and Data S1.

Both the GAD85 and NCK56 groups evidenced the enrichment of numerous gene sets related to the migration and chemotaxis of multiple types of immune cells, including neutrophils and mononuclear cells, including lymphocytes. A broad range of immune signaling pathways were found to be enriched in both groups (Figure 7A,B), including gene sets related to interleukins 1, 1β, 2, 4, 6, 8, 10, 12, and 17, TGF-β, IFN-α, IFN-β, and IFN-γ, as well as cytokines from the tumor necrosis factor superfamily. Also enriched were the TLR-2, 3, 4, and 9 signaling pathways.

**Figure 8:**
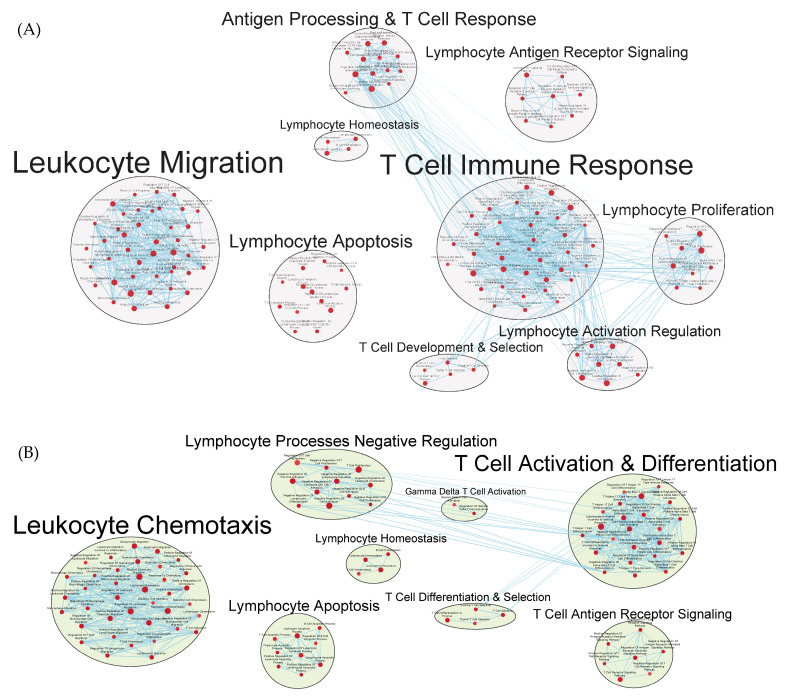
Gene sets related to T cells enriched in GAD85 compared to STI (**A**) and NCK56 compared to STI (**B**) at an FDR ≤ 0.25. Nodes represent pathways, and they are grouped within clusters based on closeness of function and shared genes represented by edges linking these nodes. Details of the within-cluster pathways, genes, FDR Q-values, and other information can be found in Tables S4 and S5 for A and B, respectively, and Data S1.

Phagocytosis and antigen processing and presentation gene sets were enhanced. Cell-mediated responses were evident by enriched gene sets related to T cell activation, differentiation, and signaling, as well as to the activities of CD4^+^ T cells and Th1 and Th17 responses (Figure 8A,B and Tables S4 and S5). Enrichment related to NK cell differentiation was observed in both the GAD85 and NCK56 groups, as well as to NK cell activation in the GAD85 group (Figure S4A,B). Both groups evidenced numerous enriched gene sets related to viral activity and the antiviral response, including host modulation of viral activities, regulation of viral processes such as transcription and replication, and cytoplasmic viral pattern recognition receptor signaling. Also notable were enriched gene sets related to inflammasome complex assembly, anoikis, tolerance induction, and the organ/tissue-specific immune response.

**Figure 9:**
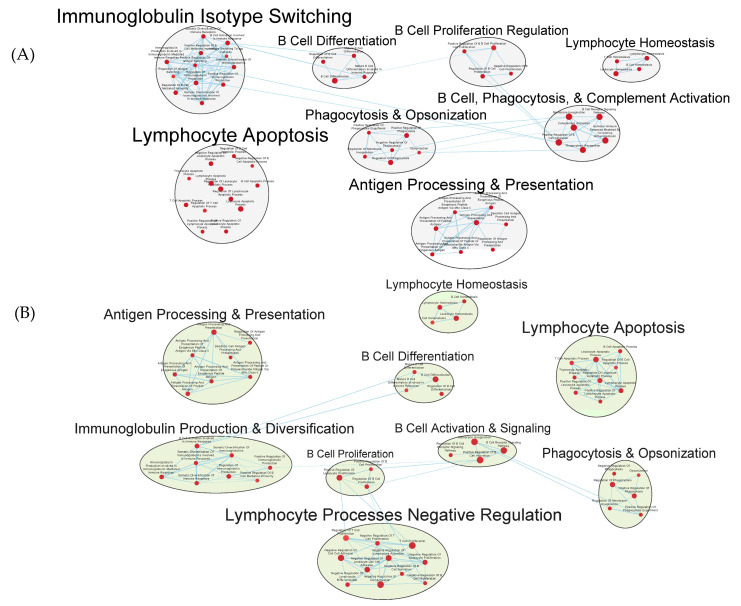
Gene sets related to B cells enriched in GAD85 compared to STI (**A**) and NCK56 compared to STI (**B**) at FDR ≤ 0.25. Nodes represent pathways and they are grouped within clusters based on closeness of function and shared genes represented by edges linking these nodes. Details of the within-cluster pathways, genes, FDR Q values and other information can be found in Tables S4 and S5, for A and B respectively, along with Data S1.

Numerous facets of the B cell response were found to be enriched, including those related to B cell activation, differentiation, and proliferation, BCR signaling, as well as to immunoglobulin production and somatic diversification (Figure 9A,B and Tables S4 and S5).

Numerous gene sets of immunological interest were enriched in the GAD85 treatment group and not in the NCK56 group when compared to the STI negative control. These included gene sets related to the antimicrobial humoral response, immunoglobulin isotype switching to IgG, CD8^+^ T cell activation, NK cell activation, and the type 2 immune response (Figures S2 and S3, Tables S4 and S5).

### 3.5. Other Gene Sets That Were Enriched between the GAD85, NCK56, and STI Groups

At an FDR value ≤ 0.25, enriched gene sets were related to physiological processes as diverse as cardiogenesis and neural development. The physiological significance of these findings is unknown. Descriptions of all gene sets enriched at the level of FDR ≤ 0.25 can be found in Table S10 for the analysis of the GAD85 group compared to the NCK56 group, Table S11 for the GAD85 group compared to the STI group, and Table S12 for the NCK56 group compared to the STI group.

## 4. Discussion

Systems biology to identify vaccine-induced immune responses that are associated with protection from infection or disease is a relatively new frontier in vaccinology. To date, these studies have primarily been focused on systemic immune responses and parenterally delivered vaccines [27,28]. Here, we described a novel model for evaluating an oral vaccine’s effects on local immune inductive sites i.e., Peyer’s patches. Utilizing transcriptomics, we evaluated the innate host immune response to an orally delivered *L. acidophilus* vaccine platform (GAD85) expressing model antigens from murine rotavirus and multiple TLR-stimulating and immune-targeting adjuvants. We also utilized metagenomics to confirm the presence of the vaccine platform within the Peyer’s Patches and metatranscriptomics to confirm its expression of the exogenous antigens and adjuvants.

Peyer’s patches are important sites of intestinal luminal sampling and antigen uptake, processing, and presentation by the resident antigen-presenting cells. We found that in both the GAD85- and NCK56-vaccinated mice, there was a clear predominance of *L. acidophilus* associated with the sampled Peyer’s patch (Figure 1). In mice receiving GAD85, we detected the expression of the antigens and immune-stimulating adjuvants (Figure 2), confirming that the rotavirus epitopes (VP8-1 and VP8Pep) and adjuvant proteins (FliC and FimH) are being actively expressed by the rLA construct. This is our first attempt to confirm and quantify such expression in vivo and highlights the utility of this method for accessing mucosal vaccines at sites of immune induction.

Transcriptomics identified several pathways that were altered in the mice immunized with NCK56 and GAD85. The transcriptomes of the GAD85 group mice have enriched gene sets related to B cell activation, BCR signaling, phagocytosis, and the humoral response when compared to the NCK56 group (Figure 6). An increase in T cell activity was identified in response to *L. acidophilus* (in both the GAD85 and NCK56 groups) delivery (Figure 9B) and was further enhanced in the GAD85 group. The CD4^+^ T cell subset appears to be enhanced in both *L. acidophilus* groups (Figure 5 and Figure 9A,B). There is also an effect by *L. acidophilus* on the NK cell response (Figure S4B). Interestingly, GAD85 group transcriptomes had enriched gene sets related to NK cell activation as well as NK cell-mediated immunity as compared to the NCK56 group (Figure S1), suggesting that the presence of *L. acidophilus* together with our vaccine antigens/adjuvants enhanced NK cell activity. There was an enrichment of gene sets related to many cytokines and immune signaling pathways, including IL-6, IL-12, and TNF-α, in the GAD85 and NCK56 groups as compared to the negative control (Figure 7A,B), but not between GAD85 and NCK56 (Figure 4). This suggests that *L. acidophilus* itself may be potentiating these immune signaling pathways. Many other immunologically relevant gene sets were also enriched in the NCK56 group compared to the STI group, including sets related to the antiviral defense, as well as a diverse variety of other innate and adaptive immune system processes (Figure S3).

Previously published studies evaluating the immune-interacting and -stimulating endogenous characteristics of lactic acid bacteria and *L. acidophilus* specifically correlate with many of the gene set changes that we identified in the NCK56 and GAD85 groups. *L. acidophilus* possesses an endogenous M cell-binding protein that allows for uptake into Peyer’s patches via binding to uromodulin, resulting in the rapid alteration of gene pathways reported here (observed here 24 h following oral delivery) [29]. The effects of lactic acid bacteria in the gastrointestinal tract on cytokine response, dendritic cell/antigen-presenting cell activation, phagocytosis, B cell subset differentiation, NK cells, T cell subsets, and anti-viral activity have previously been demonstrated [30,31]. It has been shown that different probiotic bacteria result in different mucosal transcriptome expression profiles in the human gastrointestinal mucosa, induce different proliferation responses in murine splenocytes, and can have different immunomodulatory properties and effects on cytokines [32,33,34,35]. These studies highlight the importance of an in vivo method to understand the immune response to a probiotic vaccine platform, given that the induction of adaptive immune responses and memory are likely to be affected by the probiotic strain, addition of immune-stimulating adjuvant/s, the resident microbiome, the presence of mucosal inflammation, and host pathogen load.

This study identified numerous pathways that are relevant and interesting to explore in the context of the development of a *L. acidophilus* vaccine vector. How to relate these findings to vaccine-induced protection from infection and disease is unknown and reveals current limitations in the field of vaccinology. Identification of pre- and post-immunization immune profiles or transcriptional responses from blood with the goal of identifying profiles that correlate with optimal vaccine performance is being actively studied [28,36]. A recent publication evaluated 13 different vaccines and showed that a pre-vaccination pro-inflammatory endotype that included upregulation of genes encoding for pathogen-associated molecular patterns correlated with a better response to vaccination [28]. Post-vaccination gene expression from these same individuals identified vaccines that clustered into groups based on similar immune responses, even though there was a heterogenous response between these clusters [36]. Such studies show the possibility of systems vaccinology to identify pre-vaccination states and vaccination types or adjuvants that could perform better in individuals or populations depending on age, health status, or with previous or current pathogen exposure. It is important to note that while these studies evaluated an impressive 13 vaccines, all were systemic vaccines, and their findings using peripheral blood may not be applicable to mucosal vaccines. It is recognized that mucosal immune responses may not correlate to or be predicted from the blood [37]. Additionally, the added variables of access and uptake into mucosal immune induction sites and the presence of the local microbiome, which may influence the immune response, suggest that a local evaluation of innate immune responses, as detailed here, may be best with mucosal vaccines.

In characterizing host responses to NCK56 and GAD85, we utilized Gene Set Enrichment Analysis (GSEA), which is a methodology, and accompanying software package developed to assist in the interpretation of differential expression datasets by identifying sets of genes that are enriched in the transcriptomes of one study group as compared to another. By focusing on sets rather than individual genes, this technique helps illuminate upregulated processes and pathways whose genetic signatures may be complex or too broadly distributed for accurate detection by individual gene analysis [38]. This work is exploratory in nature, and the sample size was small; thus, we set the threshold for our discussion at a discovery level of significance (FDR ≤ 0.25), which allowed us to detected patterns of gene set expression that are consistent with our expectations of immune system responses to our *L. acidophilus* construct. The immune pathways identified in this analysis all require additional confirmatory studies.

Additionally, this study is limited by the lack of evaluation of adaptive responses and immune memory. It is of great interest to the investigators that gene sets related to antigen presentation, B cell class switching, and immunoglobulin responses were identified by this method in as little as 24 h post-oral vaccine delivery. Gene sets related to these pathways were identified primarily in the GAD85 group compared to the NCK56 group. It is not possible to discern immunoglobulin subtype from these GSEA findings, but the GOBP_ORGAN_OR_TISSUE_SPECIFIC_IMMUNE_RESPONSE (q = 0.0864, NES = 1.7935) gene set enriched in the GAD85 group transcriptomes may be suggestive of the induction of the mucosal immunity component with which IgA production is typically associated. Future studies to associate these altered gene pathways with T cell activation, B cell maturation, and isotype switching are important next steps to further develop our *L. acidophilus* vaccine platform.

In conclusion, utilizing transcriptomics with GSEA, we were able to confirm the presence of our *L. acidophilus* vaccine platform at mucosal immune inductive sites and identify enriched gene sets in multiple pathways with relevance to immune activation and potentially adaptive immune responses. Further studies to confirm these findings and correlate them with protection and immune memory will help inform the continued development of our probiotic vaccine platform.

## Figures and Tables

**Table 1:** Experimental design.

Treatments	Male	Female	Total
GAD85 (recombinant NCK56 *Lactobiaccillus acidophilus* expressing dual-antigens, VP8-1 and VP810AA, and adjuvants, FimH and FliC). Associated samples were coded as V1, V2, …, V6.	3	3	6
NCK56 (wild-type NCK56 *Lactobacillus acidophilus*, positive control). Associated samples were coded as L1, L2, …, L6.	3	3	6
STI (negative control). Associated samples were coded as B1, B2, …, B5.	3	2	5
**Total**	9	8	17

## Data Availability

The sequence data are available online through NCBI’s SRA repository accession number PRJNA1120905. All other data are available either in the main manuscript or in the Supplementary Materials.

## Supplementary Materials

The following supporting information can be downloaded at: https://www.mdpi.com/article/10.3390/vaccines12070701/s1, Figure S1: Gene sets related to NK cells enriched in GAD85 compared to NCK56 at an FDR ≤ 0.25. Details of the within-cluster pathways, genes, FDR Q-values, and other information can be found in Table S3 and Data S1; Figure S2: Immunologically relevant gene sets enriched in GAD85 compared to STI at an FDR ≤ 0.25. Details of the within-cluster pathways, genes, FDR Q-values, and other information can be found in Table S4 and Data S1; Figure S3: Immunologically relevant gene sets enriched in NCK56 compared to STI at an FDR ≤ 0.25. Details of the within-cluster pathways, genes, FDR Q-values, and other information can be found in Table S5 and Data S1; Figure S4: Gene sets related to NK cells enriched in GAD85 compared to STI (A) and in NCK56 compared to STI (B) at an FDR ≤ 0.25. Details of the within-cluster pathways, genes, FDR Q-values, and other information can be found in Table S4 and S5 for panels A and B, respectively, and Data S1; Table S1: GSEA report for GAD85 in GAD85 vs. NCK56 (GESA Table); Table S2: GSEA report for NCK56 in GAD85 vs. NCK56 (GESA table); Table S3: GSEA comparison results for GAD85 vs. NCK56 at an FDR of 0.25 (Cystoscape table); Table S4: GSEA comparison results for GAD85 vs. STI at an FDR of 0.25 (Cystoscape table); Table S5: GSEA comparison results for NCK56 vs. STI at an FDR of 0.25 (Cystoscape table); Table S6: GSEA report for GAD85 in GAD85 vs. STI (GSEA table); Table S7: GSEA report for STI in GAD85 vs. STI (GSEA table); Table S8: GSEA report for NCK56 in NCK56 vs. STI (GSEA table); Table S9: GSEA report for STI in NCK56 vs. STI (GSEA table); Table S10: GSEA comparison results for GAD85 vs. NCK56 at FDR0.25 (Cystoscape table); Table S11: GSEA comparison results for GAD85 vs. STI at FDR0.25 (Cystoscape table); Table S12: GSEA comparison results for NCK56 vs. STI at an FDR of 0.25 (Cystoscape table); Data S1: Complete GSEA results.
