# Supplementary material for: Host Functional Response to a Prototypic Orally Delivered Self-Replicating Vaccine Platform
Source: Vaccines (Basel). 2024 Jun 21;12(7):701. doi: 10.3390/vaccines12070701 (PMC11281611; doi:10.3390/vaccines12070701)
Supplement: Supplementary file 1 [file vaccines-12-00701-s001.zip › Supp-Tables-and-Figures/Fig S4 GAD85vsSTI Subset NKcells FDR0.25.pdf]

A)

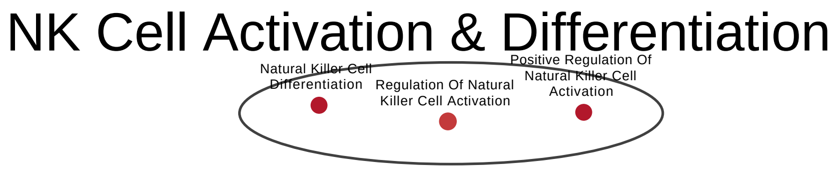

B)

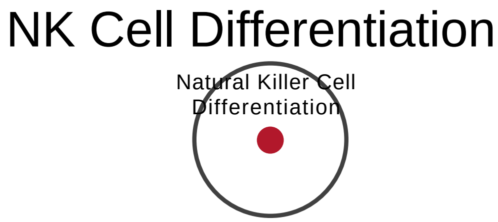

Figure 5. Gene sets related to NK cells enriched in GAD85 compared to STI (A) and NCK56 compared to STI (B) at FDR < 0.25. Details of the pathways, genes, FDR Q values and other information can be found in Table S4 and S5, for A and B respectively, along with Data S1.
