# Supplementary material for: Host Functional Response to a Prototypic Orally Delivered Self-Replicating Vaccine Platform
Source: Vaccines (Basel). 2024 Jun 21;12(7):701. doi: 10.3390/vaccines12070701 (PMC11281611; doi:10.3390/vaccines12070701)
Supplement: Supplementary file 1 [file vaccines-12-00701-s001.zip › Supp-Tables-and-Figures/Fig S1 GAD85vsNCK56 Subset NKcells FDR0.25.pdf]

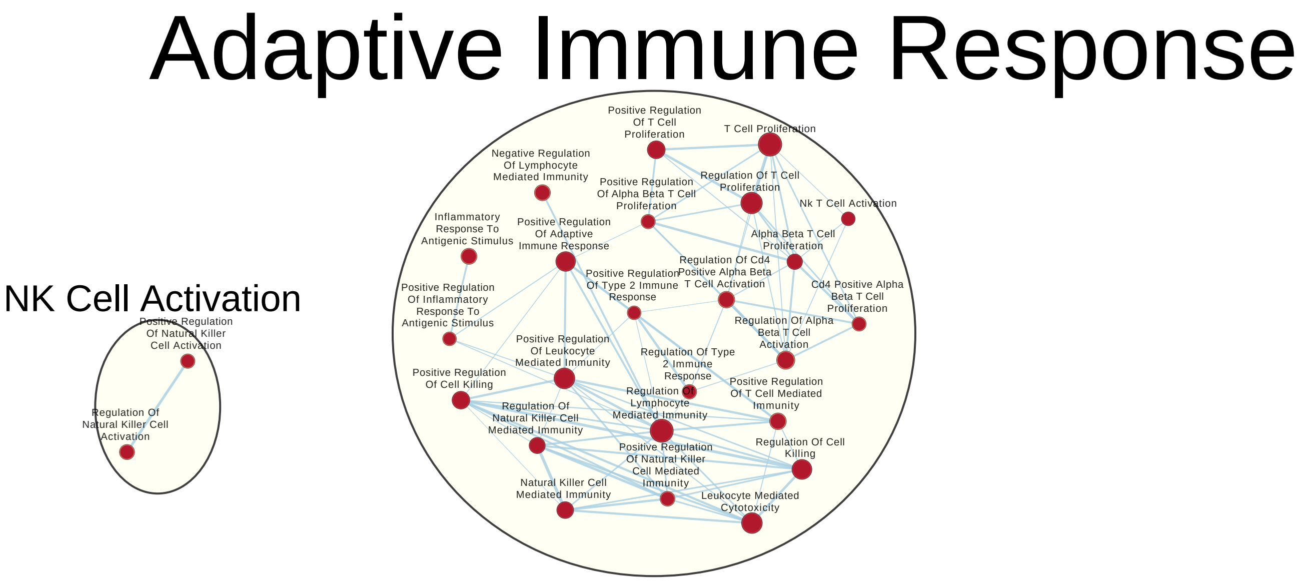

Fig. S1. Gene sets related to NK cells enriched in GAD85 compared to NCK56, at an FDR < 0.25
