# Supplementary figures and images for: Host Functional Response to a Prototypic Orally Delivered Self-Replicating Vaccine Platform
Source: Vaccines (Basel). 2024 Jun 21;12(7):701. doi: 10.3390/vaccines12070701 (PMC11281611; doi:10.3390/vaccines12070701)

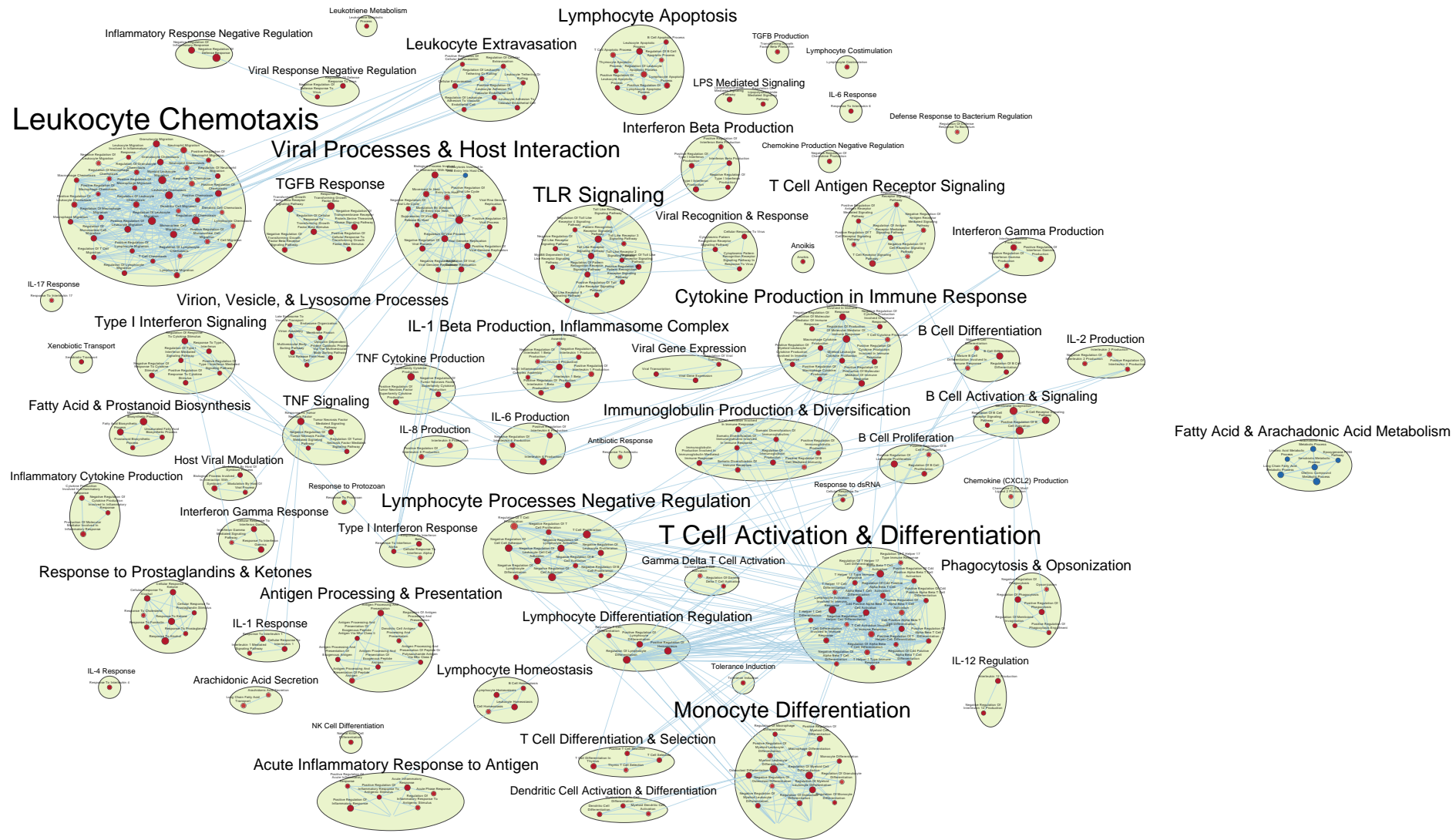

Fig. S4. Immunologically relevant gene sets enriched in NCK56 compared to BUF, at an FDR < 0.25

Supplement: Supplementary file 1 [file vaccines-12-00701-s001.zip › Supp-Tables-and-Figures/Fig S3 NCK56vsSTI Subset FDR0.25.pdf]

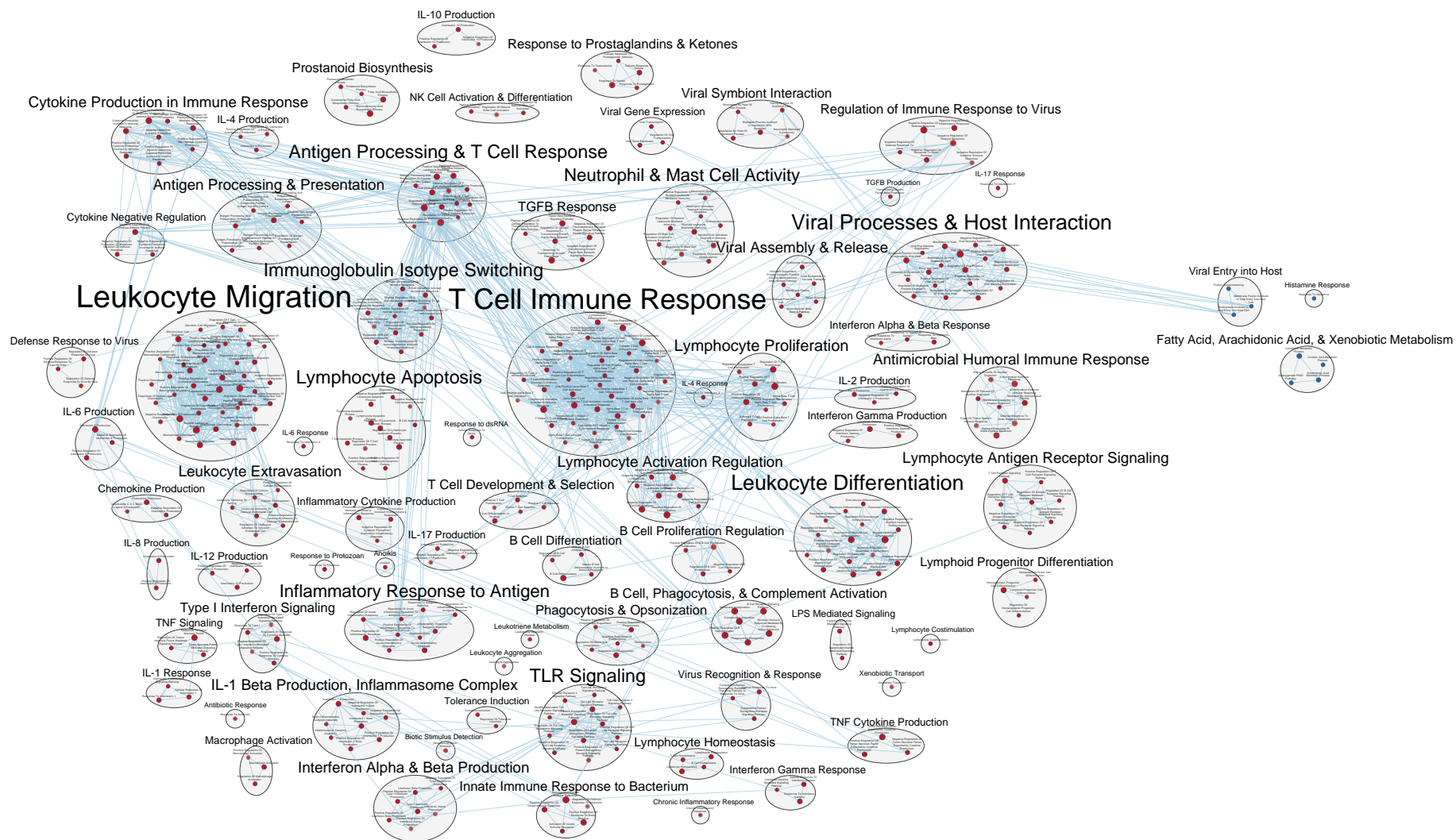

Fig. S3. Immunologically relevant gene sets enriched in GAD85 compared to BUF, at an FDR < 0.25

Supplement: Supplementary file 1 [file vaccines-12-00701-s001.zip › Supp-Tables-and-Figures/Fig S2 GAD85vsSTI Subset FDR0.25.pdf]
